# Mirror therapy in the neuroadaptive training paradigm in rehabilitation and potential mechanisms of neural remodeling: a 20-year bibliometrics analysis

**DOI:** 10.3389/fpsyg.2025.1510367

**Published:** 2025-07-30

**Authors:** Yinkai Wang, Chong Guan, Haozheng Li, Bokai Wei, Xiuqin Lv, Guohui Yang, Qian Yang, Chao Geng, Jingying Gao, Zixuan Yu, Tianbao Sun

**Affiliations:** ^1^Rehabilitation Treatment Center, The First Rehabilitation Hospital of Shanghai, School of Medicine, Tongji University, Shanghai, China; ^2^School of Rehabilitation Science, Shanghai University of Traditional Chinese Medicine, Shanghai, China; ^3^Department of Rehabilitation Medicine, Huanshan Hospital, Fudan University, Shanghai, China; ^4^Shanghai Institute of Infectious Disease and Biosecurity, Fudan University, Shanghai, China; ^5^School of Traditional Chinese Medicine, Shanghai University of Traditional Chinese Medicine, Shanghai, China; ^6^Department of Rehabilitation Medicine, Shanghai Xinqidian Rehabilitation Hospital, Shanghai, China; ^7^School of Exercise and Health, Shanghai University of Sport, Shanghai, China

**Keywords:** mirror therapy, bibliometric analysis, stroke, phantom limb pain, systematic review

## Abstract

**Objective:**

Mirror Therapy activates mirror neurons, promotes neural connection regeneration, promotes brain reorganization and motor recovery, and alleviates limb pain. This study uses bibliometric methods to analyze the research trends of Mirror Therapy, aiming to fill the current research gap and provide valuable insights and suggestions for future research.

**Methods:**

Publications related to mirror therapy from 2004 to 2024 were retrieved from the Web of Science Core Collection database. Data visualization and analysis were performed using CiteSpace, VOSviewer, and Scimago Graphica.

**Results:**

We included 728 papers in the analysis and noted an increasing trend in annual publication volume. Collaboration network analysis revealed close cooperation among scholars from these major countries and institutions. The high-frequency reference vocabulary related to mirror therapy is grouped through a clustering network, revealing the main directions and hotspots of research. Keyword burst analysis reveals current research trends, including mechanism exploration, clinical applications, and clinical treatment areas.

**Conclusion:**

A 20-year bibliometric analysis of mirror therapy literature highlights its global expansion and diversification, particularly in neurology and rehabilitation, emphasizing its importance in pain relief and post-stroke recovery, with future interdisciplinary research focusing on biomarkers and neuroimaging to refine its clinical effectiveness.

## Graphical Abstract

Mirror therapy in neuroadaptive training: a 20-year bibliometric analysis on rehabilitation and neural remodeling mechanisms. 
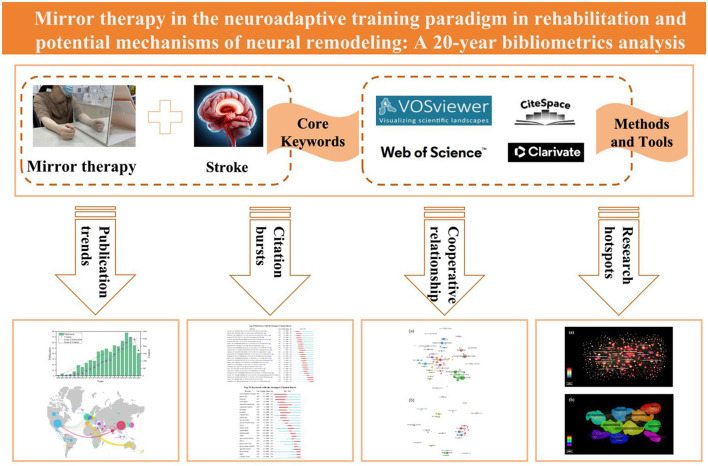

## 1 Introduction

Neural remodeling refers to the dynamic structural and functional reorganization of the nervous system in response to injury, disease, or adaptive plasticity. This process encompasses synaptic pruning, axonal regeneration, and neurogenesis, which are driven by molecular and cellular mechanisms such as inflammation, growth factor signaling, and activity-dependent plasticity. The neurorecovery process is of paramount importance in the context of numerous pathologies and traumas affecting both the central and peripheral nervous systems, with particular emphasis on traumatic brain injuries and spinal cord injuries (Turczyn et al., 2022). Understanding these mechanisms is crucial for modern healthcare, as they inform therapies such as neurorehabilitation, neuromodulation, and pharmacologic interventions aimed at enhancing recovery or mitigating maladaptive changes. The latest advancements in neuroimaging and biomarker research have significantly propelled the implementation of precision medicine strategies. Analogous to the application of molecular marker-based targeted therapies in oncology, these developments have markedly enhanced treatment outcomes and prognoses for patients with neurological disorders (Mela et al., 2023).

Mirror Therapy (MT), also known as Mirror Visual Feedback (MVF) therapy (McCabe et al., 2003), is a non-invasive treatment based on the principles of neuroplasticity. It creates a visual illusion that “trick” the brain into perceiving the affected or missing limb as present and capable of normal movement (Moseley et al., 2012). First introduced by Ramachandran and colleagues in 1995 (Ramachandran, 1996), MT was initially utilized for the treatment of neuropathic pain, including phantom limb pain and sensory deficits. Over time, its applications have expanded to encompass the rehabilitation of limb dysfunction resulting from various conditions, such as stroke, complex regional pain syndrome (CRPS), and amputations (Moseley, 2004; Ramachandran and Altschuler, 2009). While only a subset of patients experiences significant improvement, MT exhibits great potential in enhancing self-perception, restoring motor function, and reducing pain, particularly when considering the high incidence and disability rates associated with these conditions (Ramachandran and Altschuler, 2009).

MT activates mirror neurons, which are crucial for understanding and replicating motor actions (Molla-Casanova et al., 2024). The mechanism of MT likely involves the stimulation of cortical areas responsible for motor input, thereby promoting the regeneration of neural connections between the body and the cortex (Ramachandran and Altschuler, 2009; Rizzolatti and Craighero, 2004). Approximately 65% of stroke patients do not achieve satisfactory hand function recovery (Dobkin, 2005). MT addresses this issue by providing continuous visual feedback, which includes elements of movement observation, thereby stimulating the primary motor cortex, influencing cortical excitability, and reorganizing sensorimotor circuits to promote brain reorganization and motor recovery (Altschuler et al., 1999). For patients experiencing limb pain, MT compensates for the loss of somatosensory information in the affected areas by relaying this feedback to the central nervous system, thereby reducing pain. In recent years, an increasing number of researchers have been exploring the mechanisms underlying MT's efficacy in treating other conditions (Wylde et al., 2017; Bruehl, 2015). Given its safety and convenience (Ramachandran and Altschuler, 2009), MT has garnered increasing attention in recent years.

Bibliometrics is a field that employs mathematical and statistical methods to quantitatively analyze scientific literature (Ninkov et al., 2022). This approach is increasingly applied to the study of rehabilitation techniques, including Constraint-Induced Movement Therapy (CIMT) and Sensory Integration Therapy (SIT) (Xu et al., 2024; Li et al., 2023). However, trends in MT research have not yet been widely reported. This paper aims to address this gap by conducting a bibliometric analysis of studies on MT, uncovering key research topics, trends, and existing challenges, and providing valuable insights and recommendations for future research.

## 2 Materials and methods

### 2.1 Data sources and search strategy

We utilized the Web of Science Core Collection (WoSCC) to compile comprehensive and accurate publication data. By refining our search strategy, we improved both the coverage and precision of the data. To avoid issues such as duplication, missing data, or inconsistent topics, we conducted data filtering and standardization prior to analysis to ensure data quality. Our search strategy was as follows: TS = (“Mirror Therapy” OR “Mirror Visual Feedback” OR “mvf” OR “Mirror Training” OR “Mirror Illusion” OR “Mirror Box” OR “Virtual Reality Reflection Therapy” OR “VRRT”). This search was executed on August 21, 2024, resulting in 1,466 hits. After excluding non-article and non-review types, 1,370 articles were retained for analysis.

### 2.2 Inclusion criteria

**Figure 1:**
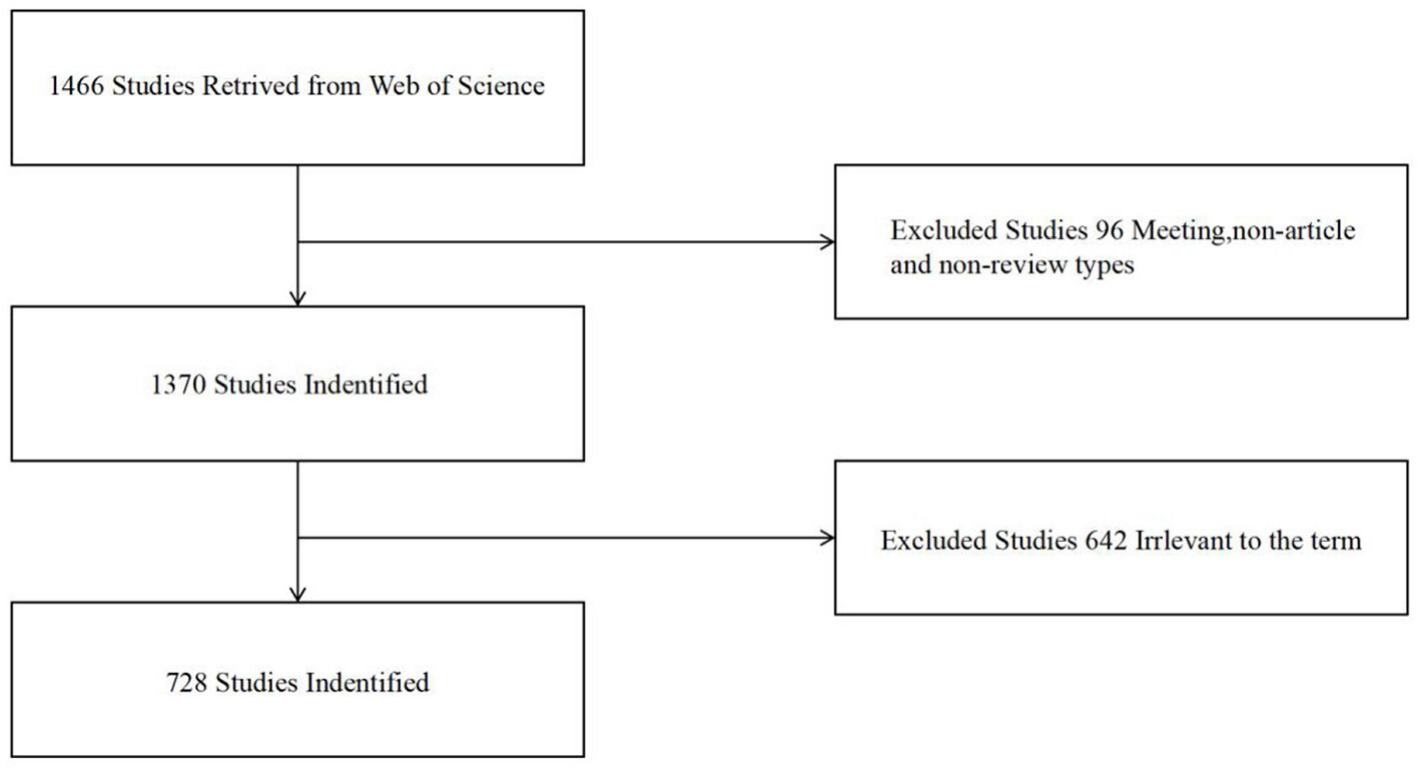
Flowchart of study inclusion criteria.

The inclusion criteria specified that articles must explicitly mention interventions or mechanisms related to MT. Following a comprehensive screening process, 642 articles were excluded for not aligning with our research focus. This resulted in a final dataset of 728 records, comprising 574 original research articles and 154 review articles, including 10 highly cited works. The review and screening process was rigorous, involving two independent team members to ensure fairness and accuracy. Discrepancies were resolved through team voting, thereby establishing a solid foundation for our analysis (Figure 1).

### 2.3 Data standardization

To account for changes in institutional or country names over the past 20 years, we standardized the names in the selected literature. We also standardized keywords to minimize inconsistencies between singular and plural forms and reduce unnecessary duplication. For instance, in this study, the keywords “meta-analysis,” “Meta Analysis,” and “meta analyses” were consolidated under the single form “meta-analysis.”

### 2.4 Data analysis

We conducted a comprehensive bibliometric analysis employing CiteSpace (version 6.4 R1) and VOSviewer (version 1.6.18) to examine key characteristics such as publication volume (van Eck and Waltman, 2010; Synnestvedt et al., 2005; Li et al., 2025), geographical distribution, institutional affiliations, author contributions, journal sources, document types, and keywords. Both VOSviewer and CiteSpace are widely recognized as robust bibliometric tools, each possessing unique strengths. VOSviewer, a freely available program, excels in visually representing large, easily interpretable bibliometric maps. In contrast, CiteSpace, also freely available, is distinguished by its capacity to uncover research hotspots, delineate developmental trajectories, and forecast future trends. The settings for CiteSpace were configured as follows: log-likelihood ratio (LLR), time slices (2000–2022), slice length (1 year), term source (all selected), selection criteria (g-index: k = 25), and pruning (pathfinder, pruning sliced networks).

## 3 Results

### 3.1 Annual publication and citation trends

**Figure 2:**
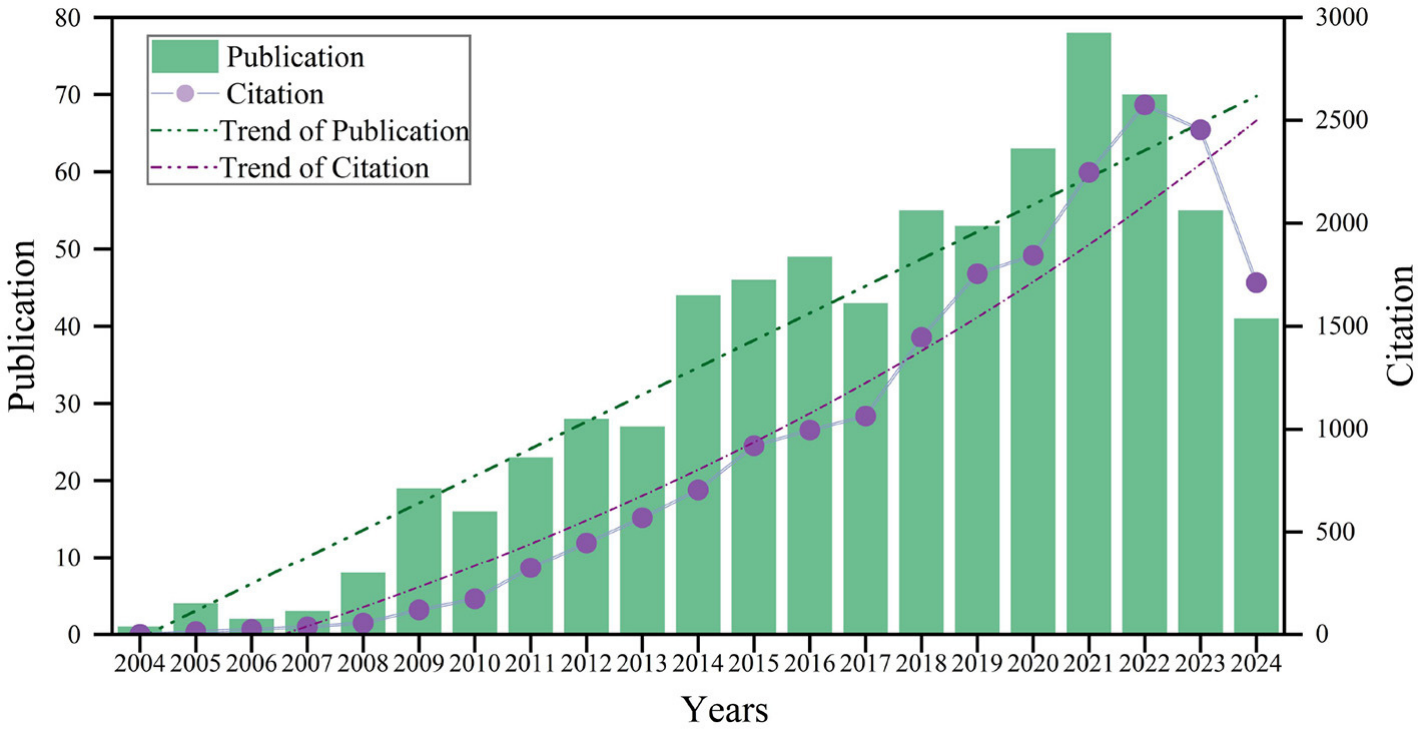
Annual publications on mirror therapy from 2004 to 2024.

The number of publications and citation frequency over specific time periods accurately reflect the pace and trends of research within the field of MT. From 2004 to 2024, the overall number of publications exhibited an upward trend, despite some annual fluctuations (Figure 2). Citation counts also increased, with a notable rise between 2017 and 2022, followed by a slight decline. In total, 728 relevant papers were published, accumulating 19,469 citations.

### 3.2 Journal analysis

#### 3.2.1 Published journals analysis

We analyzed the top 10 journals in terms of publication volume (Table 1). Among these, three journals were based in the United States, the United Kingdom, and Switzerland, respectively. The articles were primarily published in “*Frontiers in Neurology*”, “*Neurorehabilitation and Neural Repair*”, and the “*Archives of Physical Medicine and Rehabilitation*”. Notably, “*Frontiers in Neurology*” had the highest number of publications, with 26 articles, whereas “*Neurorehabilitation and Neural Repair*” received the most citations, with 1,449 citations.

**Table 1:** Top 10 published journals.

**Rank**	**Journal**	**Publications**	**Citations (WoS)**	**Average citation/publication**	**Impact Factor (2023)**	**H-index**
1	Frontiers in Neurology	26	315	12.12	2.7	9
2	Frontiers in Human Neuroscience	22	389	17.68	2.4	11
3	Neurorehabilitation and Neural Repair	19	1,449	76.26	3.7	16
4	Disability and Rehabilitation	18	353	19.61	2.1	7
5	European Journal of Physical and Rehabilitation Medicine	16	243	15.19	3.3	8
6	Clinical Rehabilitation	15	315	21.00	2.6	10
7	Brain Sciences	14	39	2.79	2.7	5
8	Archives of Physical Medicine and Rehabilitation	13	954	73.38	3.6	11
8	PLoS One	13	150	11.54	2.9	9
8	Scientific Reports	13	95	7.31	4.3	5

#### 3.2.2 Cited journals analysis

We analyzed the top 10 journals based on citation count (Table 2). Among these, two journals had an impact factor greater than 8.5: *Brain* (10.6) and *Cochrane Database of Systematic Reviews* (8.8). Furthermore, these journals are categorized within the fields of rehabilitation medicine, clinical neurology, and neuroscience, with nine of the ten ranked in Q1.

**Table 2:** Top 10 cited journals.

**Rank**	**Journal**	**Citations**	**IF (2023)**
1	Neurorehabilitation and Neural Repair	1,431	3.7
2	Archives of Physical Medicine and Rehabilitation	1,372	3.6
3	Pain	1,302	5.9
4	Brain	908	10.6
5	Stroke	792	7.8
6	NeuroImage	755	4.7
7	Experimental Brain Research	719	1.7
8	Journal of Neuroscience	586	4.4
9	Cochrane Database of Systematic Reviews	529	8.8
9	Clinical Rehabilitation	529	2.6

### 3.3 Cooperative relationship network

#### 3.3.1 Countries analysis

Upon conducting a detailed statistical analysis of the publication counts and their impact from various countries, regions, and key research institutions within the field of MT (Table 3), we uncovered the collaboration patterns and trends prevailing in this domain. China emerged as the leading country in terms of publication volume, contributing 137 papers that were cited a total of 1,748 times. The United States, on the other hand, led in terms of citations with 3,988, placing second in publication volume with 117 papers. When evaluating the average number of citations per article, Australia topped the list with an average of 71.92 citations, despite ranking ninth in terms of total publications.

**Table 3:** Top 10 productive countries/regions.

**Rank**	**Country**	**Publications**	**Citations**	**Average citations/publication**
1	China	137	1,748	12.76
2	USA	117	3,988	34.09
3	United Kingdom	84	3,904	46.48
4	Germany	70	2,877	41.10
5	South Korea	51	889	17.43
6	Italy	50	1,717	34.34
6	Japan	50	1,017	20.34
8	Spain	43	581	13.51
9	Australia	37	2,661	71.92
10	Netherlands	34	1,932	56.82

#### 3.3.2 Cooperative relationship analysis

**Figure 3:**
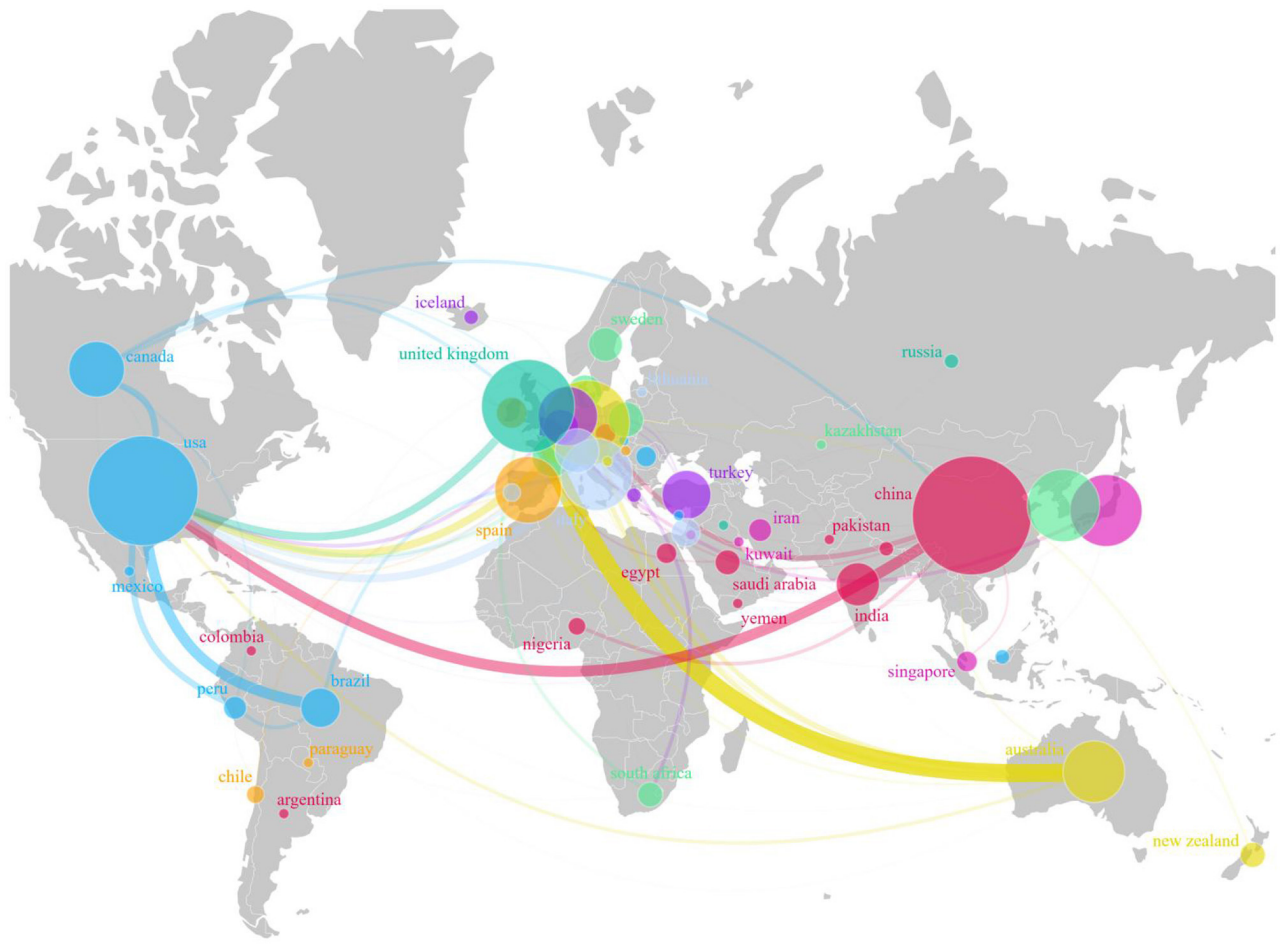
Network of international cooperation as a geographic visualization.

The standardized country collaboration map indicates that China closely collaborates with the United States, India, and other countries (Figure 3). The United States collaborates with Brazil, Canada, and Peru, which results in high publication and citation rates. Although the United Kingdom primarily collaborates with the United States, its partnerships are limited; however, it still maintains a strong publication and citation record.

#### 3.3.3 Institutional and authors analysis

**Figure 4:**
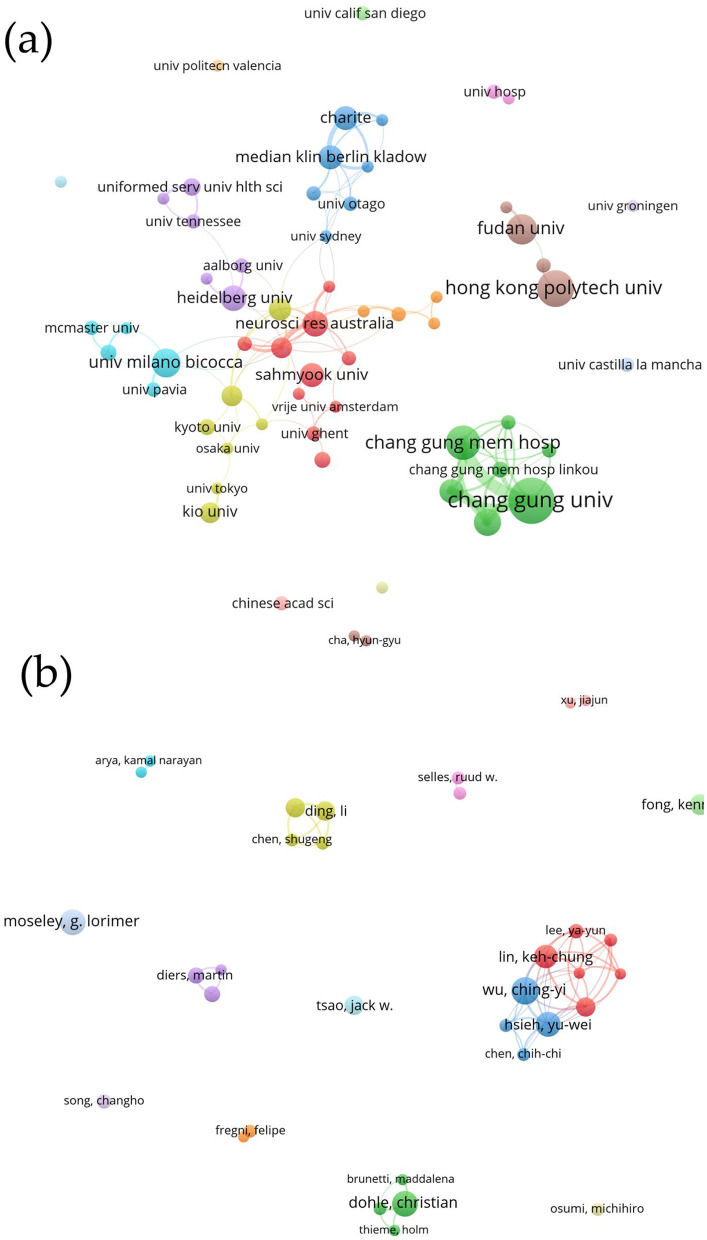
Network of international cooperation among: **(a)** Institutions; **(b)** Authors.

According to the publication statistics and VOSviewer analysis (Table 4), Chang Gung University ranked first with 28 publications, receiving 417 citations and an average of 14.89 citations per paper. Although the Florey Institute of Neuroscience and Mental Health in Australia published only 13 papers, it had the highest citation count at 1,146, with an average of 88.15 citations per paper. Among the top ten institutions, six are located in China. In terms of institutional collaboration, there is a strong interconnection and cooperative dynamic among the leading institutions in publication volume (Figure 4a).

**Table 4:** Top 10 published institutions.

**Rank**	**Institution**	**Publications**	**Citations**	**Average citations/publication**
1	Chang Gung University	28	417	14.89
2	The Hong Kong Polytechnic University	21	306	14.57
3	Chang Gung Memorial Hospital	19	182	9.58
4	Fudan University	16	201	12.56
5	University of Milano-Bicocca	15	404	26.93
6	National Taiwan University	14	295	21.07
7	Heidelberg University	13	656	50.46
7	Neuroscience Research Australia	13	1,146	88.15
9	charite	12	457	38.08
9	MEDIAN Klinik Berlin Kladow	12	566	47.17
9	National Taiwan University Hospital	12	267	22.25
9	Sahmyook University	12	315	26.25

Upon analyzing publication counts and VOSviewer data, several authors with the highest number of publications were identified (Table 5), including Wu Ching-Yi, Moseley, Lorimer, Hsieh Yu-wei, Lin Keh-chung, Fong Kenneth N.K., Flor Herta, Dohle Jack W., Cao Jack Jiaqi, Selles Ruud, and Jie. Notably, Moseley and Selles exhibited significantly higher citation counts, with 1,618 and 968 citations, respectively, and achieved high h-indices of 14 and 10. Dohle and Flor each published 11 papers, with citation counts of 638 and 633, indicating the high quality of their work, as reflected in their respective h-indices of 11. Additionally, Wu, Hsieh, Lin, and Lee Ya-yun frequently collaborate on publications. Dohle often works with Morkisch and Fritzsch, while Guo, XiaoLi, Ding Li, and Wang Hewei have also engaged in collaborative efforts. Diers, Martin, Flor, and Petrini Laura have collaborated on MT research. Other authors, such as Moseley, Xu Jisjun, and Osumi, tend to conduct independent research. While there are strong collaborations among various scholars, most are confined to single countries, with international cooperation being relatively less common (Figure 4b).

**Table 5:** Top 10 authors.

**Rank**	**Authors**	**Publications**	**Citations**	**Average citations/publication**	**H-index**
1	Moseley, Lorimer	17	1,618	95.18	14
1	Wu, Ching-Yi	17	362	21.29	10
3	Hsieh, Yu-wei	15	201	13.40	9
4	Lin, Keh-chung	13	288	22.15	9
5	Dohle, Christian	11	638	58.00	11
5	Flor, Herta	11	633	57.55	11
5	Tsao, Jack w.	11	403	36.64	9
5	Fong, kenneth N. K.	11	234	21.27	8
9	Selles, Ruud	10	968	96.80	10
9	Zhang, Jack Jiaqi	10	206	20.60	7
9	Jia, Jie	10	90	9.00	6

### 3.4 Analysis of cited and co-cited references

In terms of citation frequency, the article titled “Interventions for Improving Upper Limb Function After Stroke,” published in 2014 in the Cochrane Database of Systematic Reviews, had an impact factor of 8.8 and received 550 citations, with Alex Pollock as the first author (Table 6). This article is highly relevant to our study. The second most cited article is “Using Visual Feedback, Particularly Mirror Visual Feedback, to Restore Brain Function,” published in Brain in 2009, which has been cited 431 times and has an impact factor of 10.6, with V.S. Ramachandran as the first author. Another notable article is “Complex Regional Pain Syndrome,” published in 2015 in *BMJ-British Medical Journal*. Although it ranks eighth in citation count, it has the highest impact factor among the top ten articles at 93.6. The third most cited article is “Graded Motor Imagery is Effective for Persistent Complex Regional Pain Syndrome: A Randomized Controlled Trial,” published in Pain in 2004, which has received 408 citations.

**Table 6:** Top 10 citation articles.

**Rank**	**Citations**	**Title**	**First author**	**Years**	**Journal**	**IF (2023)**
1	550	Interventions for improving upper limb function after stroke	Pollock, Alex	2014	Cochrane Database of Systematic Reviews	8.8
2	431	The use of visual feedback, in particular mirror visual feedback, in restoring brain function	Ramachandran, V. S.	2009	Brain	10.6
3	408	Graded motor imagery is effective for long-standing complex regional pain syndrome: a randomized controlled trial	Moseley, GL	2004	Pain	5.9
4	316	Mirror therapy improves hand function in subacute stroke: A randomized controlled trial	Yavuzer, Ganes	2008	Archives of Physical Medicine and Rehabilitation	3.6
5	312	Bodily illusions in health and disease: Physiological and clinical perspectives and the concept of a cortical 'body matrix'	Moseley, G. Lorimer	2011	Neuroscience and Biobehavioral Reviews	7.5
6	271	Mirror Therapy Promotes Recovery From Severe Hemiparesis: A Randomized Controlled Trial	Dohle, Christian	2009	Neurorehabilitation and Neural Repair	3.7
7	262	Motor Recovery and Cortical Reorganization After Mirror Therapy in Chronic Stroke Patients: A Phase II Randomized Controlled Trial	Michielsen, Marian E.	2011	Neurorehabilitation and Neural Repair	3.7
8	241	Complex regional pain syndrome	Bruehl, Stephen	2015	Bmj-British Medical Journal	93.6
9	239	Postamputation pain: epidemiology, mechanisms, and treatment	Hsu, Eugene	2013	Journal of Pain Research	2.5
10	204	Efficacy of Upper Limb Therapies for Unilateral Cerebral Palsy: A Meta-analysis	Sakzewski, Leanne	2014	PEDIATRICS	6.2

When two articles are cited together by a third article, this phenomenon is known as co-citation. Co-citation indicates a close relationship and relevance between the cited works, highlighting their interconnectedness within a research field. Such articles often contain high-quality content that has a significant impact on their respective areas of study. Moreover, the relationships among co-cited works are dynamic and can evolve over time, reflecting the changing nature of development within specific disciplines. The most frequently co-cited article is “Phantom Limb Phenomena Induced by Mirrors” by V.S. Ramachandran, published in 1996 in Proceedings of the Royal Society B: Biological Sciences, with 206 citations (Table 7). Ramachandran also authored two other co-cited articles: “Using Visual Feedback, Particularly Mirror Visual Feedback, to Restore Brain Function,” published in Brain in 2009 (159 citations), and “The Perception of Phantom Limbs,” published in Nature in 1995 (158 citations), ranking third and fifth in co-citation frequency, respectively. Ranking second is “Rehabilitation of Hemiparesis After Stroke” by E.L. Altschuler, published in The Lancet in 1999, with 175 citations. Gunes Yavuzer's article, “Mirror Therapy Improves Hand Function in Subacute Stroke Patients: A Randomized Controlled Trial,” published in Physical Therapy and Rehabilitation Archives in 2008, ranks fourth with 159 citations. It is also noteworthy that “Mirror Therapy for Phantom Pain” has fewer citations, but it was published in The New England Journal of Medicine, which has a high impact factor of 96.2.

**Table 7:** Top 10 co-citation articles.

**Rank**	**Citations**	**Title**	**First author**	**Year**	**Journal**	**IF (2023)**
1	206	Synaesthesia in phantom limbs induced with mirrors	V S Ramachandran	1996	Proceedings of The Royal Society B-biological Sciences	3.8
2	175	Rehabilitation of hemiparesis after stroke with a mirror	E L Altschuler	1999	Lancet	98.4
3	159	The use of visual feedback, in particular mirror visual feedback, in restoring brain function	V S Ramachandran	2009	Brain	10.6
4	159	Mirror therapy improves hand function in subacute stroke: a randomized controlled trial	Gunes Yavuzer	2008	Archives of Physical Medicine And Rehabilitation	3.6
5	158	Touching the phantom limb	V S Ramachandran	1995	Nature	50.5
6	153	Mirror therapy for phantom limb pain	Brenda L Chan	2007	New England Journal of Medicine	96.2
7	151	Mirror therapy promotes recovery from severe hemiparesis: a randomized controlled trial	Christian Dohle	2009	Neurorehabilitation and Neural Repair	3.7
8	135	Motor recovery and cortical reorganization after mirror therapy in chronic stroke patients: a phase II randomized controlled trial	Marian E. Michielsen	2011	Neurorehabilitation and Neural Repair	3.7
9	126	Mirror, mirror on the wall: viewing a mirror reflection of unilateral hand movements facilitates ipsilateral M1 excitability	M I Garry	2005	Experimental Brain Research	1.7
10	122	Reflections on mirror therapy: a systematic review of the effect of mirror visual feedback on the brain	Frederik J A Deconinck	2015	Neurorehabilitation and Neural Repair	3.7

### 3.5 Citation burst analysis

**Figure 5:**
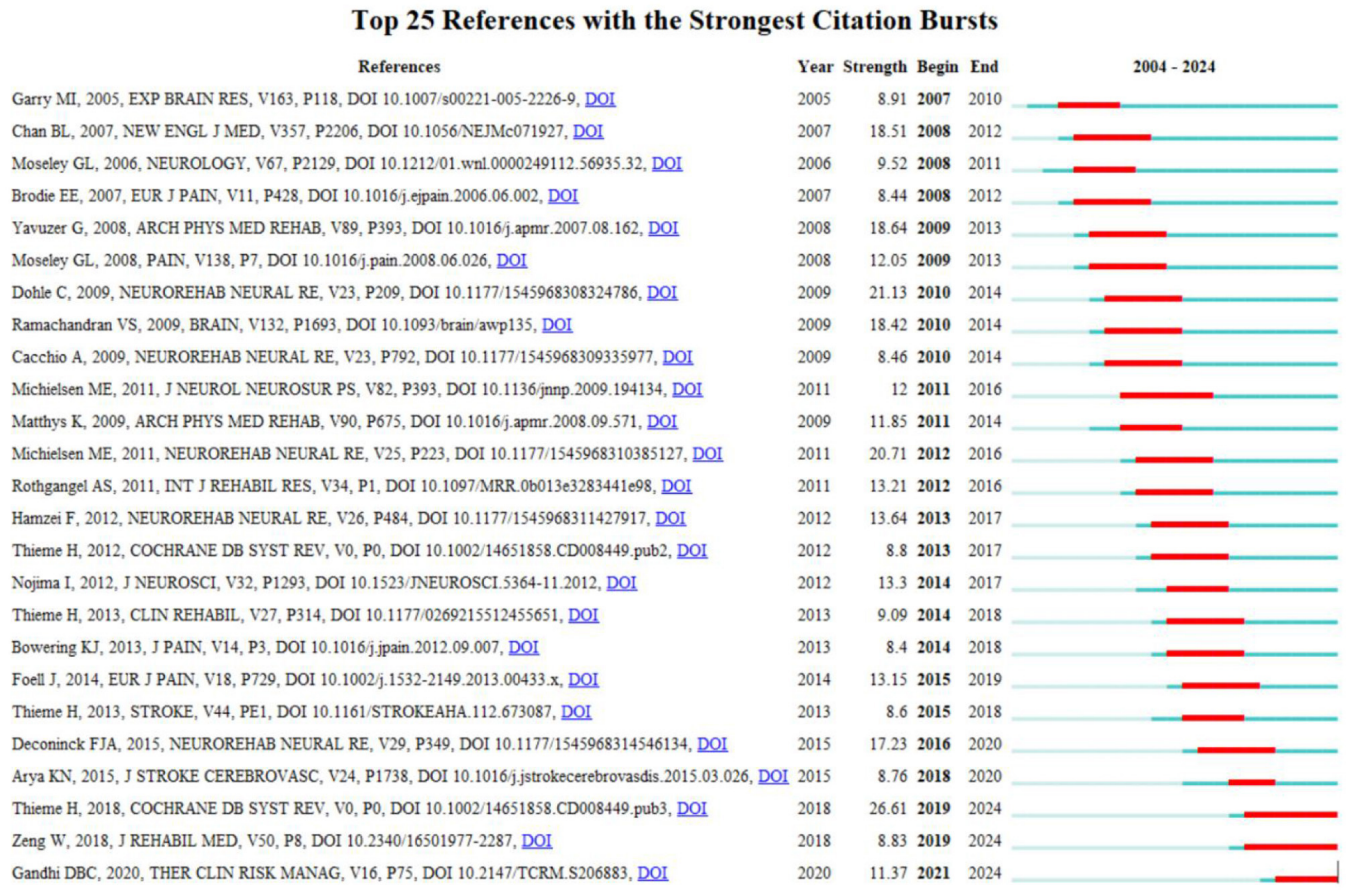
Top 25 references with the strongest citation bursts during 2004 to 2024.

The term “citation burst” refers to a phenomenon in which an article experiences a sudden increase in the frequency of citations following its publication. This occurrence is significant for identifying influential literature from a particular time period. The blue line represents the timeline, with red segments denoting detected citation bursts, indicating the start year, end year, and duration of each burst. The three articles with the highest citation burst strengths are as follows: “Mirror Therapy for Improving Motor Function After Stroke,” which exhibited a burst strength of 26.61 from 2019 to 2024; “Mirror Therapy Promotes Recovery From Severe Hemiparesis: A Randomized Controlled Trial,” with a burst strength of 21.13, primarily between 2010 and 2014; and “Motor Recovery and Cortical Reorganization After Mirror Therapy in Chronic Stroke Patients: A Phase II Randomized Controlled Trial,” with a burst strength of 20.71, mainly from 2012 to 2016 (Figure 5).

### 3.6 Keyword analysis

**Figure 6:**
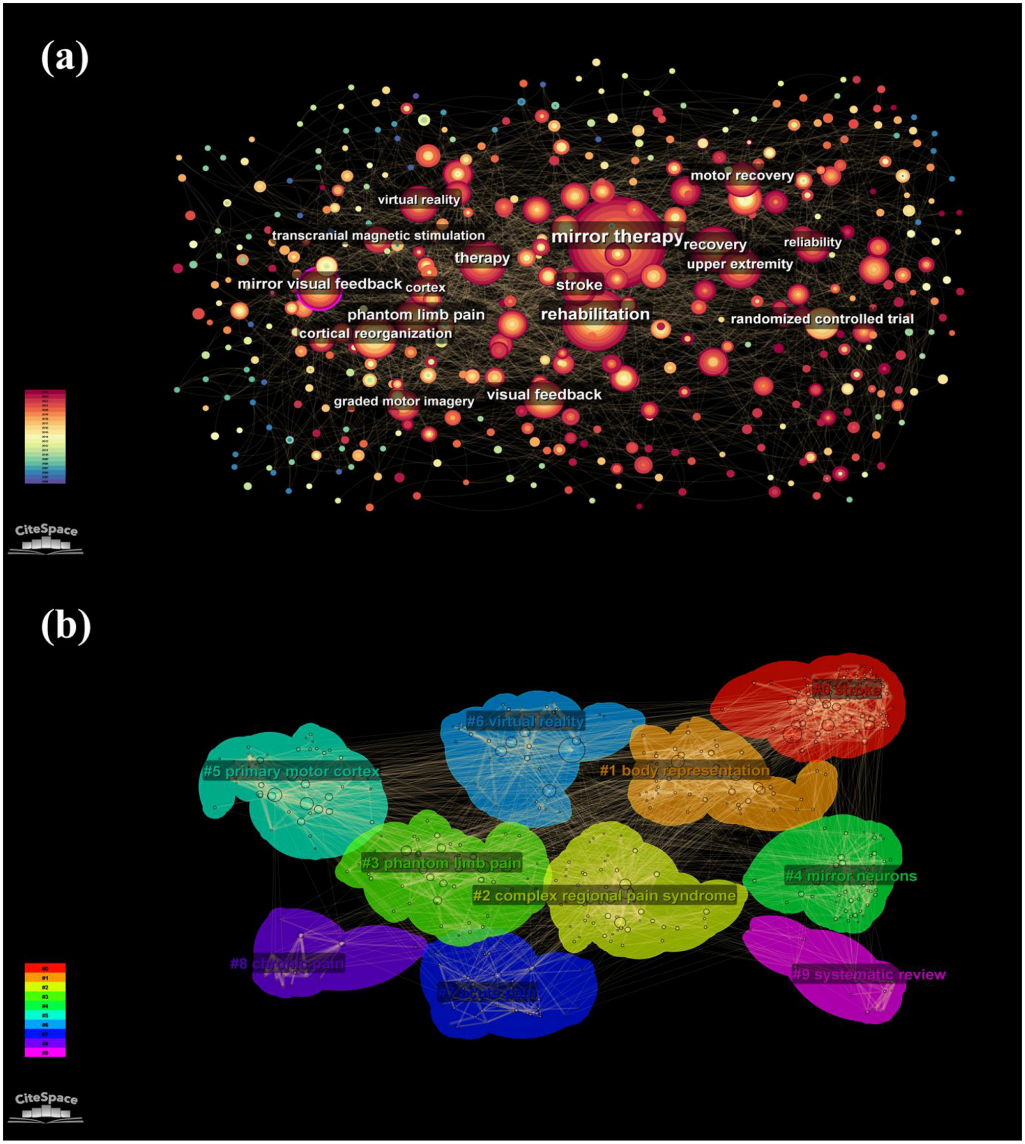
Keywords of: **(a)** Frequency network; **(b)** Clustering network.

Keywords encapsulate the essence of a publication, summarizing its primary research focus. The co-occurrence of these terms reveals key research trends and hot topics within a specific field. The top ten frequently cited keywords related to MT are “mirror therapy” (367 citations), “rehabilitation” (271), “stroke” (268), “phantom pain” (113), “treatment” (112), “mirror visual feedback” (101), “recovery” (99), “visual feedback” (94), “randomized controlled trial” (91), “upper limb” (87), “graded motor imagery” (Pan et al., 2024), and “cortex” (Jo et al., 2024) (Figure 6a). To further analyze these keywords, we organized and categorized them using a clustering network. The keyword co-occurrence graph illustrates the concentration and frequency of these terms, with the size of each node proportional to its total citation frequency (Figure 6b). The co-occurring keywords were divided into ten subgroups: stroke, body representation, complex regional pain syndrome, phantom limb pain, mirror neurons, primary motor cortex, virtual reality, acute pain, chronic pain, and systematic review.

**Figure 7:**
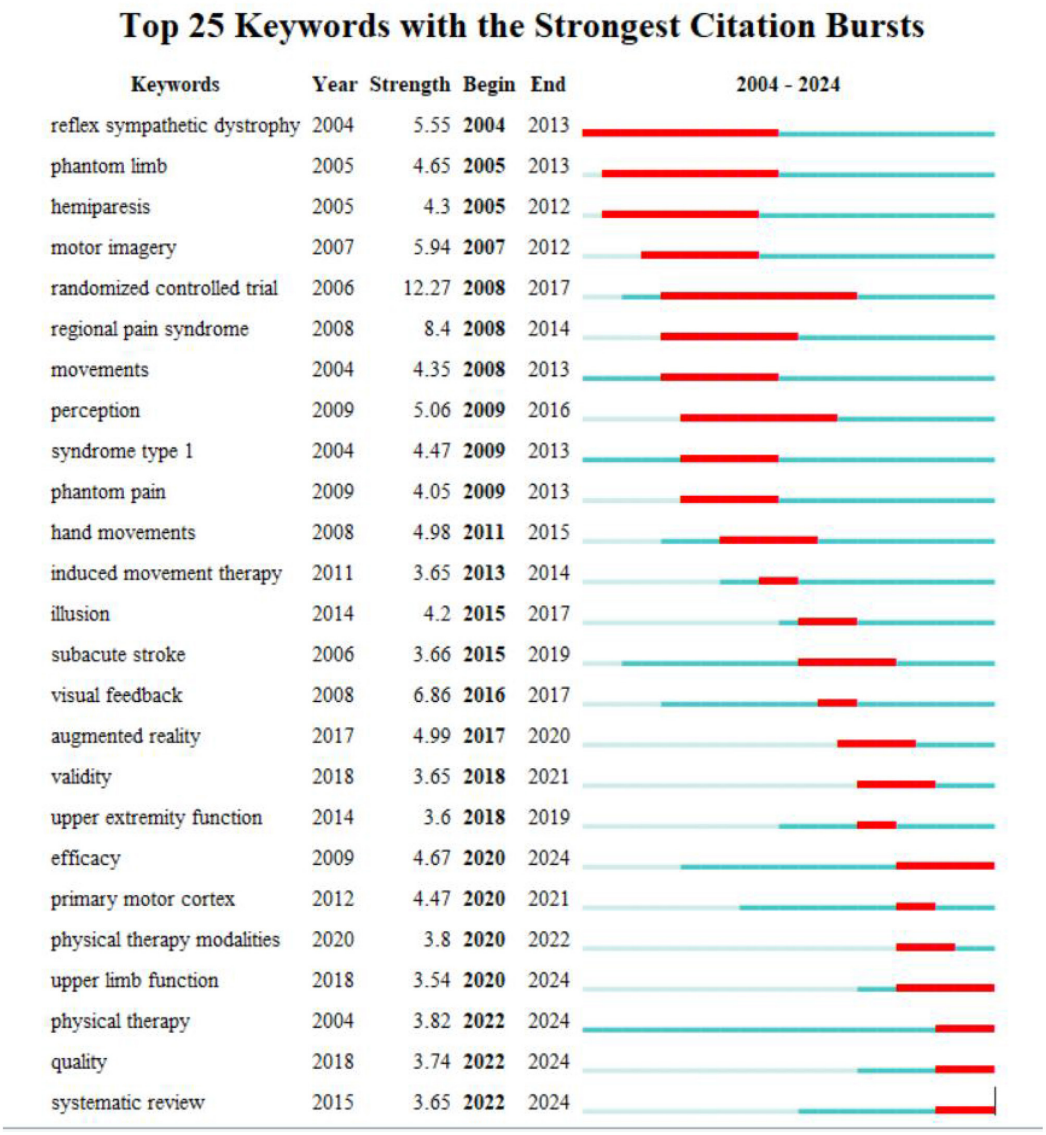
Top 25 keywords with the strongest citation bursts during 2004 to 2024.

Similar to citation bursts, keyword bursts signify a sharp increase in the frequency of specific terms within published academic articles. This phenomenon aids in identifying prevailing research trends during certain periods. The keyword with the highest burst strength is “randomized controlled trial” (12.27), followed by “regional pain syndrome” (8.4), “visual feedback” (6.86), “motor imagery” (5.94), and “reflex sympathetic dystrophy” (5.55) (Figure 7). Notably, certain keywords, such as “efficacy,” “upper limb function,” “physical therapy,” “quality,” and “systematic review,” remain in their burst phase. These keywords can be categorized into three main groups: Mechanism exploration, including “visual feedback” and “motor imagery”; Clinical application, encompassing “systematic review” and “randomized controlled trial”; and Clinical treatment areas, involving “regional pain syndrome,” “upper limb function,” and “reflex sympathetic dystrophy.”

## 4 Discussion

MT is a widely used clinical intervention known for its effectiveness in alleviating phantom limb pain post-amputation and in aiding the recovery of motor and sensory functions post-stroke (Deconinck et al., 2015). Its ease of implementation, safety, and non-invasive nature have contributed to its international recognition and adoption. This study employs bibliometric analysis and network visualization to examine the research landscape of MT over the past two decades. By analyzing the contributions of various countries, institutions, journals, and authors, this study highlights emerging trends and predicts future research directions within the field.

### 4.1 General information

Between 2004 and 2021, the annual publication volume on MT exhibited a steady increase. The relatively low output from 2004 to 2007 likely reflects the nascent stage of research in this area, with limited attention from researchers. Interest surged following a 2007 study published in The New England Journal of Medicine, which demonstrated the effectiveness of MT in reducing phantom limb pain (Chan et al., 2007). Over the subsequent 2 years, the number of publications increased nine-fold compared to 2007. From 2017 to 2022, citation rates rose significantly, possibly due to a 2016 systematic review that highlighted previous research limitations and proposed new directions (Barbin et al., 2016). The slight decline in research growth from 2021 to 2023 may be attributed to the impact of COVID-19 on clinical trials. As of July 2024, 41 papers had been published, and research output is expected to rebound by the end of the year.

### 4.2 Research status

#### 4.2.1 Published and cited journals

Among the top ten journals for publications on MT, eight are ranked in Q1 and Q2, indicating widespread research and application in fields such as rehabilitation medicine, clinical neuroscience, and neurology. Furthermore, nine of the top ten most-cited journals are Q1-ranked, reflecting the high level of interest and recognition in these areas. The interdisciplinary application of MT has enhanced the dissemination of research findings, promoted collaboration among experts, and strengthened the integration of theory and practice.

#### 4.2.2 Influential countries and institutions

China leads the field in terms of publication output, being the only developing country among the top contributors, followed by the United States. The high volume of publications may be attributed to China's large population and the increasing incidence of stroke. Interestingly, China's inaugural article in this domain was published as recently as 2012, evaluating the efficacy of MT in enhancing upper limb motor function post-stroke (Toh and Fong, 2012). Since then, domestic researchers have actively engaged in clinical trials to explore the applications of MT for motor and sensory function recovery. Nevertheless, China's research in this area is still nascent, which may account for the relatively low average citation count per article. Australia, despite ranking ninth in terms of publication volume, boasts the highest average citation rate. This could be due to the influence of Professor Moseley, a preeminent expert in the field, whose early reviews published in high-impact journals have attracted considerable academic attention (Moseley et al., 2012, 2008; O'Connell et al., 2013; Bowering et al., 2013). Most research on MT originates from developed countries, benefiting from superior resources and enhanced opportunities for collaboration. It is worth noting that Chang Gung University and Chang Gung Memorial Hospital rank first and third in terms of output, highlighting the robust synergy between academic and clinical practice in these institutions. However, collaboration between countries, institutions, and authors remains limited. While countries such as China, Japan, and South Korea have produced significant research, international cooperation is still inadequate. Increasing collaborative efforts, particularly in countries with lower cooperation rates, could facilitate the sharing of insights, foster opportunities, and ultimately advance the field, improving overall research quality.

#### 4.2.3 Authorship and collaboration

Among the top ten most published authors in the field, three—Professors Wu Ching-Yi, Hsieh Yu-wei, and Lin Keh-chung—have collaborated closely on studies examining the clinical effects of MT on motor, sensory, and daily functional recovery in stroke patients (Wu et al., 2013; Lin et al., 2014a; Hsieh et al., 2020; Lin et al., 2014b). Another research group, led by Professors Christian Dohle, Nadine Morkisch, and Claire Fritzsch from MEDIAN Klinik Berlin Kladow, has focused on exploring the neurobiological mechanisms underlying MT (Wang et al., 2013a,b; Fritzsch et al., 2014; Dohle et al., 2011). Interestingly, although Professor Moseley, a key figure in pain research, does not have consistent collaborators, his work has attracted widespread attention and citations. This could be attributed to the strong overlap between his research on pain and the therapeutic applications of MT (Moseley and Butler, 2015).

#### 4.2.4 Cited and co-cited references' direction

Citation analysis reveals the evolving research trends in the field of MT. The most cited paper over the past 20 years is a 2014 review by Professor Alex Pollock, published in the Cochrane Database of Systematic Reviews. This article highlights the current state of interventions for upper limb recovery after stroke, emphasizing the need for more high-quality randomized controlled trials (RCTs) to confirm the efficacy of MT (Pollock et al., 2014). The prominence of this journal underscores the importance of publishing in high-impact journals to drive further exploration and increase citation rates.

Notably, four of the top ten most-cited articles in this field are RCTs (Moseley, 2004; Yavuzer et al., 2008; Dohle et al., 2009; Michielsen et al., 2011a). These high-quality trials have enhanced the reliability and credibility of MT research. The remaining six articles are review studies, which have provided a solid theoretical foundation for the application of MT, while also identifying existing challenges and gaps in clinical practice (Moseley et al., 2012; Ramachandran and Altschuler, 2009; Bruehl, 2015; Pollock et al., 2014; Hsu and Cohen, 2013; Sakzewski et al., 2014). This combination of clinical trials and reviews has enabled researchers to design more targeted experimental studies to address these gaps and provide stronger scientific evidence for clinical use. Overall, these findings suggest that MT, as an emerging rehabilitation technique, has demonstrated significant potential and is gradually gaining recognition and maturity within the scientific and medical communities.

#### 4.2.5 Global trend of MT research

Citation surges, defined as a rapid increase in the number of references cited by papers within a specific period, highlight global research trends in MT over time. Between 2007 and 2010, research by Professor Garry MI demonstrated that observing mirrored movements of an unaffected limb could stimulate the ipsilateral motor cortex (Garry et al., 2005), providing neurophysiological evidence for the therapeutic use of MT in rehabilitation, this visual feedback process aids in rewiring the brain and promotes functional recovery. Building on this foundation, Professor Nojima and colleagues later used transcranial magnetic stimulation (TMS) to confirm that the ability of MT to improve motor function is closely linked to the remapping of M1, particularly in regions with heightened neuronal excitability (Nojima et al., 2013). Between 2008 and 2012, a study led by Professor Chan BL and published in the New England Journal of Medicine found that MT could alleviate phantom limb pain, potentially by activating mirror neurons in the hemisphere opposite the amputated limb (Chan et al., 2007). Similarly, Professor Rizzolatti and colleagues suggested that mirror neurons play a crucial role in action observation, intention understanding, movement imitation, learning, and motor imagery (Rizzolatti and Craighero, 2004). Although the role of mirror neurons in these processes has been widely acknowledged, definitive evidence of long-term neural changes remains limited. From 2012 to 2016, Professor Marian E. Michielsen utilized functional MRI to explore whether MT induces cortical reorganization. The results indicated that MT could stimulate a shift in M1 activation toward the damaged hemisphere, suggesting neural reorganization. However, a six-month follow-up did not include measurements of cortical activation, leaving the persistence of these changes unverified (Michielsen et al., 2011b).

Between 2016 and 2020, Professor Frederik J. A. Deconinck reviewed various potential mechanisms by which MT may influence brain function, identifying key areas for further research (Deconinck et al., 2015). The majority of the evidence originated from studies on healthy adults, whereas those involving patient populations primarily focused on stroke survivors. The potential of MT to impact multiple functional networks highlights its promise as an intervention to support motor recovery; however, further research is required to fully understand its effects on the brain.

Large-scale clinical trials, including those assessing brain function and structure, aim to evaluate the efficacy of MT across diverse populations and to elucidate the underlying mechanisms, as well as to identify potential group differences. Although further research is required to fully comprehend and harness the potential of MT in neurorehabilitation, its substantial impact on motor system regulation is irrefutable. Regrettably, recent studies have predominantly focused on the recovery of upper limb motor function post-stroke, with diminished attention to the exploration of the underlying mechanisms. This shift in focus may be attributed to two primary factors. Firstly, the COVID-19 pandemic has impeded numerous studies investigating these mechanisms. Secondly, the increasing incidence of stroke and the inadequate recovery of hand function in many stroke survivors have heightened the demand for clinical solutions. Consequently, research has prioritized practical applications, potentially at the cost of more profound investigation into the therapy's mechanisms.

### 4.3 Research hotspots and frontiers of MT

The analysis of keyword frequency, clustering, and citation bursts reveals three primary research areas in the field of MT: targeted conditions, underlying mechanisms, and clinical applications.

#### 4.3.1 Diseases treated by MT

The first cluster of studies concentrates on conditions targeted by MT, including stroke, CRPS, phantom limb pain, chronic pain, and acute pain. This highlights the therapy's extensive clinical applications, as researchers aim to understand how MT can improve neural function recovery in damaged areas by modulating brain plasticity.

##### 4.3.1.1 Phantom limb pain and chronic pain

After amputation, many patients experience a persistent sensation associated with the lost limb, with 60% to 90% of amputees suffering from phantom limb pain (Foell et al., 2014). This persistent and chronic pain poses a significant challenge to treatment. A notable increase in the frequency of the keyword “phantom limb” from 2005 to 2013 likely correlates with the introduction of MT by Professor Ramachandran in 1995, which was applied to patients with phantom limb pain (Ramachandran, 1996). Subsequent RCTs, including those conducted by Professor Brenda, reinforced the effectiveness of this approach. They hypothesized that MT could alleviate pain in these patients, exerting a significant influence within the medical community (Chan et al., 2007). In 2014, Professor Foell's research further revealed that the effectiveness of MT varies among patients with phantom limb pain, with only some experiencing significant benefits (Foell et al., 2014). This finding underscores the necessity for personalized treatment strategies. Investigating individual differences may be crucial for understanding the mechanisms of MT. Future research should focus on the brain network activity patterns in patients to identify more precise treatment indicators.

##### 4.3.1.2 Stroke

As research progresses, an increasing number of disease areas are exploring the application of MT. Around the same time, Professor Altschuler published findings in The Lancet in 1999 (Altschuler et al., 1999), introducing MT for stroke patients and emphasizing the keyword “hemiparesis,” Professor Yavuzer demonstrated significant improvements in upper limb motor function among subacute stroke patients. This intervention was shown not to exacerbate spasticity (Yavuzer et al., 2008). In contrast, Professor Dohle indicated that restoring upper limb function in severe stroke patients presents a significant challenge; however, early intervention with MT may enhance sensory and motor functions (Dohle et al., 2009). From 2015 to 2019, there was a notable increase in the frequency of the keyword “subacute stroke,” likely due to heightened interest among researchers and a deeper understanding of the rehabilitation window following a stroke. During this period, the brain exhibits considerable plasticity, which MT can leverage to facilitate neural recovery. Despite ongoing debates, numerous studies continue to support the potential of MT in improving motor function and reducing pain.

In recent years, there has been a growing focus on the broader applications of MT. Since 2020, with the rising incidence of strokes and the corresponding demand for effective rehabilitation strategies, research has increasingly centered on “upper limb function.” The challenge of restoring hand function is expected to dominate future studies in this field.

##### 4.3.1.3 Complex regional pain syndrome

CRPS predominantly affects one side of the body and is characterized by prolonged, often unbearable burning pain (Bruehl, 2015). The pathophysiology of CRPS is not fully understood, and identifying effective treatments poses a challenge. Between 2008 and 2014, the unique neurophysiological effects of MT garnered attention for its potential in CRPS research. Studies by Professor Cacchio demonstrated that MT significantly alleviated upper limb pain in patients with CRPS type I (Cacchio et al., 2009).

However, some studies have expressed skepticism regarding the effectiveness of MT in pain relief (Shafiee et al., 2023). In 2024, Professor Machac conducted a RCT that revealed significant positive effects of MT in reducing pain among CRPS patients. Additionally, slight improvements were observed in chronic CRPS type I patients with a disease duration exceeding 12 months (Machac et al., 2024). This finding offers a more encouraging perspective for patients who have long suffered from pain than previously suggested by Professor McCabe (McCabe et al., 2003).

The application of MT in the treatment of CRPS is gradually providing new insights and possibilities for clinical practice.

##### 4.3.1.4 Acute pain

Acute pain presents a significant global medical challenge throughout the treatment process (Clark and Horton, 2018). Research conducted by Professor Brun Clementine indicates that the distinction between acute and chronic pain may arise from parietal lobe dysfunction in chronic pain patients, whereas acute pain is associated with heightened activation in this brain region (Brun et al., 2017). This difference can occasionally result in a temporary exacerbation of acute pain during the use of MT.

In response to this issue, recent studies have begun to explore the potential benefits of combining MT with other rehabilitation methods. Over the past few years, researchers have assessed the effects of non-invasive brain stimulation (NIBS) in conjunction with behavioral therapy for treating acute pain (Zhao et al., 2022). They found that NIBS enhanced the activation of mirror neuron system (MNS) of MT by regulating cortical excitability, forming a synergistic effect. This provides a new choice for patients with drug resistance or rehabilitation platform stage. In this context, a study by Professor Mariana Agostinho found that the combined application of transcranial direct current stimulation (tDCS) and MT significantly reduced early acute phantom limb pain, with lasting effects (Agostinho et al., 2023). However, the existing clinical trials focus on patients in the chronic phase, and lack of systematic evaluation of the combination therapy in the acute or subacute phase (such as the rTMS combined with tDCS within 0-6 weeks after stroke).

This underscores the importance of preventing neuroregulatory disturbances in acute pain and sets the stage for the integration of MT with other treatment modalities in the future.

#### 4.3.2 Potential mechanisms of MT

The second cluster of research concentrates on the underlying mechanisms of MT, particularly the roles of mirror neurons and the primary motor cortex. Researchers are seeking to understand the functional mechanisms of mirror neurons and their interactions with the primary motor cortex.

##### 4.3.2.1 Mirror neurons

The discovery of the MNS stands as one of the most significant advancements in neuroscience over the past few decades (Rizzolatti and Craighero, 2004). MNS is a specialized neural network initially discovered in the macaque premotor and parietal cortices, later corroborated in humans through neuroimaging and electrophysiological studies. This system exhibits bimodal activation properties, firing both during: action execution (e.g., grasping an object) and action observation (e.g., watching another individual perform the same grasp). These specialized neurons activate not only during the execution of conscious movements but also when observing the actions of others, triggering similar electrical signals (Zhang et al., 2024). This phenomenon calls for a deeper exploration of how the brain comprehends and replicates behaviors. MT harnesses this system by activating mirror neurons and engaging the corresponding motor pathways, thereby enhancing attention to the affected limb and facilitating functional recovery (Zhang et al., 2024). The coordinated activation of bilateral mirror neuron systems, motor networks, and attention networks facilitates the seamless operation of these pathways. Research by Professor Dong Wei suggests that robot-assisted virtual reality MT may provide a more effective means of activating the mirror neuron system (Wei et al., 2022). However, before this approach can be widely applied in clinical settings, a series of high-quality studies are needed for further validation and refinement. Despite the insights gained, the specific role of mirror neurons in acute pain remains unclear. Future studies must investigate how these neurons regulate pain perception and their precise contributions to alleviating discomfort.

##### 4.3.2.2 Primary motor cortex

The neural networks of the brain are intricately connected, with some motor neurons originating from the healthy hemisphere and projecting to the affected hemisphere (Deconinck et al., 2015). These motor pathways are crucial for restoring motor function in impaired limbs. The application of TMS has revealed the potential of MT to stimulate activity in the primary motor cortex or enhance activity in the damaged M1 region during unilateral hand movements (Rjosk et al., 2017). Recent research using magnetoencephalography (MEG) has explored how MVF influences cortical activity in stroke patients during unilateral and bilateral hand movements (Shih et al., 2017). This research demonstrates that both movement patterns can activate the impaired primary motor cortex, promoting functional recovery in the brain. Professor Qiu Yaxian utilized synchronous near-infrared spectroscopy to compare the immediate hemodynamic cortical activation in healthy subjects engaged in four different visual feedback tasks (Qiu et al., 2022). The findings indicated that the combination of MT with a mechanical bilateral rehabilitation system resulted in greater cortical activation than either method alone. Additionally, during MT, motor imagery tasks activated the premotor cortex on the contralateral side. Furthermore, Waller et al. observed through fMRI that bilateral symmetric movements led to increased activation in the motor cortex (Waller et al., 2014). This suggests that MT may help streamline some motor pathways on the impaired side, thereby facilitating the recovery of limb motor function. These findings further underscore the critical role of MT in promoting brain functional reorganization.

#### 4.3.3 The actual clinical application of MT

##### 4.3.3.1 Randomized controlled trials

High-quality RCTs enhance the reliability of research and support advancements in clinical treatment. In recent years, many RCTs have integrated MT into comprehensive intervention designs. For instance, a 2018 study by Kim et al. demonstrated that high-frequency repetitive transcranial magnetic stimulation (HF-rTMS) combined with task-oriented motor therapy effectively improved hand function in acute stroke patients (Kim and Yim, 2018).

Recent studies, including Gebreheat's research in 2024, have found that the combination of virtual reality with MT safely and effectively enhances upper limb function and engagement in stroke patients (Gebreheat et al., 2024). Furthermore, Jo's study from the same year indicated that a 360-degree immersive virtual reality, when combined with MT, outperformed traditional physical therapy in improving upper limb function, engagement, and motivation among subacute stroke patients (Jo et al., 2024). Similarly, Pan Hong's research in 2024 highlighted the positive effects of combining MT with electrical stimulation on the daily living abilities of subacute stroke patients (Pan et al., 2024).

These findings underscore the multifaceted potential of MT in promoting sensory and motor recovery. While most RCTs demonstrate the efficacy of MT, there remains a need for larger, multicenter clinical trials to further validate its long-term effectiveness and safety.

##### 4.3.3.2 Systematic review

Systematic reviews provide valuable insights into the current state, limitations, and future directions of MT across various conditions. A significant challenge in many RCTs of MT is their small sample sizes, which complicate the drawing of definitive conclusions regarding their effectiveness compared to other therapies. Comprehensive systematic reviews and meta-analyses are therefore essential (Samuelkamaleshkumar et al., 2014; Simpson et al., 2019; Sütbeyaz et al., 2007).

In recent years, numerous systematic reviews and meta-analyses have examined the direct effects of MT on motor function, addressing aspects such as balance, walking speed, upper limb movement, daily living activities, spasticity, and sensory impairments (Muñoz-Gómez et al., 2023). These reviews not only provide theoretical support for MT but also highlight issues within clinical practice. Such insights will guide future research to more effectively address existing gaps and facilitate the clinical application of MT.

## 5 Limitations

Our research inevitably has certain limitations. Firstly, as MT is a relatively specialized and emerging field, the existing body of literature remains limited in scope, which may have influenced the breadth of our analytical perspective. Secondly, the study was primarily based on the Science Web database, and while this provided valuable insights, it might not fully capture the complete academic landscape of the field. Furthermore, the inclusion of both articles and reviews in our analysis, while providing a broader overview, may introduce some nuances when interpreting citation-based impact metrics. It is also worth noting that some noteworthy recent publications might not yet have gained significant citation traction due to their more recent publication dates. Future research should aim to address these limitations to provide a more comprehensive understanding of the field.

## 6 Conclusion

We conducted a bibliometric analysis of literature on MT over the past 20 years to provide an initial understanding of its global research landscape and trends. Our findings indicate that the field of MT has significantly expanded and diversified. The research highlights an increasing relevance of MT in neurology and rehabilitation, particularly in alleviating pain caused by neurological conditions and enhancing recovery post-stroke. The interdisciplinary nature of this research connects neuroscience, rehabilitation, and sports medicine, underscoring the complexity and vast potential of MT. It is anticipated that future investigations will focus on the mechanisms of MT from interdisciplinary perspectives, including biomarkers and neuroimaging. As we continue to explore the potential of MT, we remain hopeful that our efforts will further clarify the effectiveness and refinement of this neuroadaptive training paradigm in clinical practice and sports settings.

## Data Availability

The datasets presented in this article are not readily available because not applicable. Requests to access the datasets should be directed to Yinkai Wang, wangyinkai0412@163.com.
